# *N*-[4-(9-Chloro­quino[3,2-*b*]benzo[1,4]thia­zin-6-yl)but­yl]acetamide^1^

**DOI:** 10.1107/S1600536812045680

**Published:** 2012-11-10

**Authors:** Małgorzata Jeleń, Kinga Suwińska, Krystian Pluta, Beata Morak-Młodawska

**Affiliations:** a Department of Organic Chemistry, The Medical University of Silesia, ul. Jagiellońska 4, 41-200 Sosnowiec, Poland; b Institute of Physical Chemistry, Polish Academy of Sciences, ul. Kasprzaka 44/52, 01-224 Warsaw, Poland; c Faculty of Biology and Environmental, Sciences, Cardinal Stefan Wyszynski University, ul. Wóycickiego 1/3, PL-01 938, Warszawa, Poland

## Abstract

In the title mol­ecule, C_21_H_20_ClN_3_OS, the tetra­cyclic system is close to planar [r.m.s. deviation = 0.110 (4) Å]. The dihedral angle between the quinoline ring system and the benzene ring is 178.3 (1)° and the angle between two (S—C=C—N) halves of the thia­zine ring is 173.4 (1)°. In the crystal, mol­ecules are arranged *via* π–π inter­actions [centroid–centroid distances = 3.603 (2)–3.739 (2) Å] into slipped stacks extending along [010]. Inter­molecular N—H⋯O hydrogen bonds link the amide groups of neighbouring mol­ecules along the stack, generating a *C*(4) motif. The title compound shows promising anti­proliferative and anti­cancer activity.

## Related literature

For recent literature on biological activity of phenothia­zines, see: Aaron *et al.* (2009 ▶); Pluta *et al.* (2011 ▶). For the synthesis and biological activity of 6-substituted quinobenzothia­zines, see: Jeleń & Pluta (2009 ▶); Pluta *et al.* (2012 ▶). For the folded structures of similar tetra­cyclic systems, see: Jeleń *et al.* (2012 ▶); Luck *et al.* (2003 ▶); Yoshida *et al.* (1994 ▶). For crystal structures of phenothia­zines, see: Chu (1988 ▶). For information on aza­phenothia­zines, and their nomenclature and synthesis, see: Pluta *et al.* (2009 ▶).
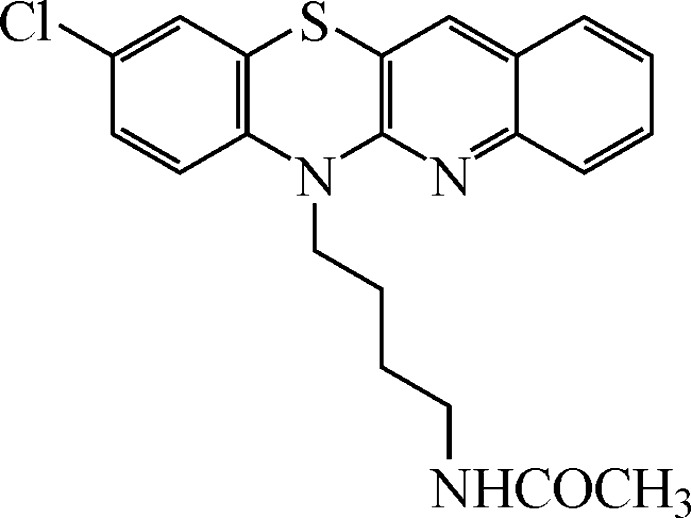


## Experimental

### 

#### Crystal data

C_21_H_20_ClN_3_OS
*M* *_r_* = 397.92
Monoclinic, 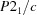

*a* = 12.7800 (4) Å
*b* = 4.9530 (11) Å
*c* = 28.781 (2) Å
β = 97.726 (5)°
*V* = 1805.3 (4) Å^3^
*Z* = 4
Mo *K*α radiation
μ = 0.35 mm^−1^
*T* = 100 K
0.60 × 0.10 × 0.05 mm

#### Data collection

Nonius KappaCCD diffractometer upgraded with an APEXII detector
17434 measured reflections
3032 independent reflections
1987 reflections with *I* > 2σ(*I*)
*R*_int_ = 0.121

#### Refinement

*R*[*F* ^2^ > 2σ(*F* ^2^)] = 0.067
*wR*(*F* ^2^) = 0.127
*S* = 1.10
3032 reflections
245 parameters
H-atom parameters constrained
Δρ_max_ = 0.32 e Å^−3^
Δρ_min_ = −0.29 e Å^−3^

### 

Data collection: *COLLECT* (Nonius, 1998 ▶); cell refinement: *DENZO* and *SCALEPACK* (Otwinowski & Minor, 1997 ▶); data reduction: *DENZO* and *SCALEPACK*; program(s) used to solve structure: *SHELXS97* (Sheldrick, 2008 ▶); program(s) used to refine structure: *SHELXL97* (Sheldrick, 2008 ▶); molecular graphics: *ORTEPIII* (Burnett & Johnson, 1996 ▶) and *Mercury* (Macrae *et al.*, 2008 ▶); software used to prepare material for publication: *publCIF* (Westrip, 2010 ▶).

## Supplementary Material

Crystal structure: contains datablock(s) I, global. DOI: 10.1107/S1600536812045680/gk2524sup1.cif

Structure factors: contains datablock(s) I. DOI: 10.1107/S1600536812045680/gk2524Isup2.hkl

Additional supplementary materials:  crystallographic information; 3D view; checkCIF report

## Figures and Tables

**Table 1:** Hydrogen-bond geometry (Å, °)

*D*—H⋯*A*	*D*—H	H⋯*A*	*D*⋯*A*	*D*—H⋯*A*
N18—H18⋯O21^i^	0.88	1.97	2.819 (4)	163

Symmetry code: (i) 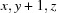.

**Fig. 1.**
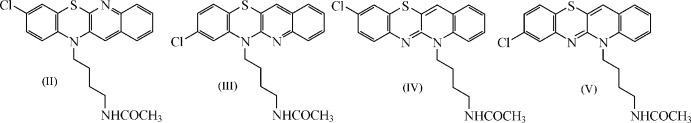
Alternative structures of the title compound.

**Fig. 2.**
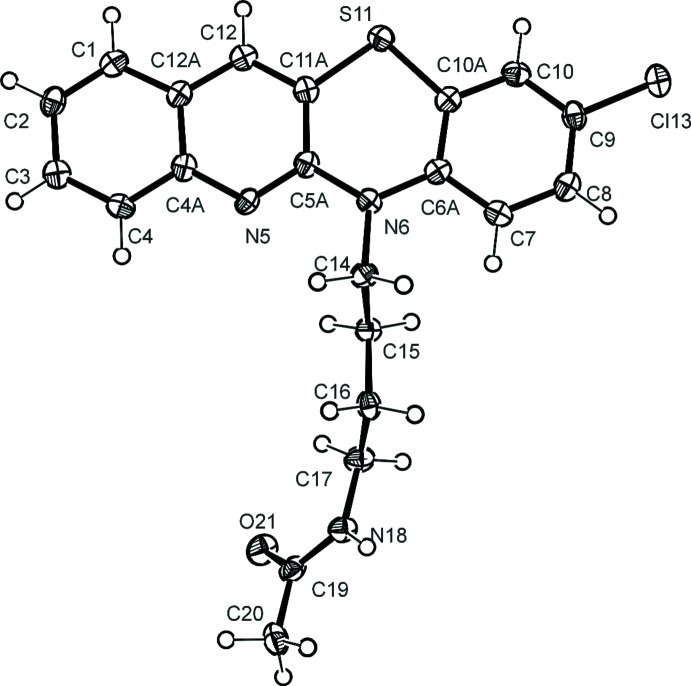
ORTEP drawing with displacement ellipsoids shown at the 50% probability level. SmilesCrystal packing shown along the b crystallographic axis.

**Fig. 3.**
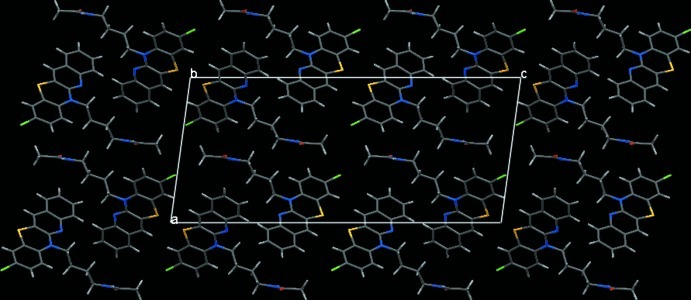
π–π stacking of the aromatic rings and one dimensional hydrogen-bond network.

**Fig. 4.**
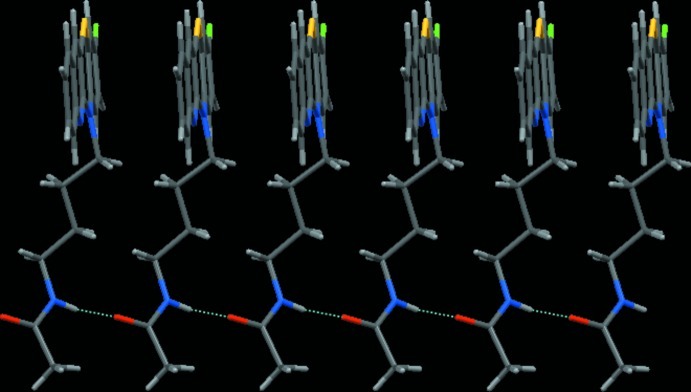
?

## supplementary crystallographic information

### Comment

Classical phenothiazines are widely recognized as neuroleptic, antihistaminic and antitissive drugs. New phenothiazines are obtained by the introduction of new pharmacophoric substituents at the thiazine nitrogen atom and the substitution of the benzene ring with an azine ring (Pluta *et al.*, 2009, 2011). Both classical and newly synthesized phenothiazines exhibit valuable anticancer, antibacterial and reversal multidrug resistance (Aaron *et al.*, 2009; Pluta *et al.*, 2011). We modified the phenothiazine structure *via* the substitution of the benzene ring with the quinoline ring to form linear fused quinobenzothiazines. The title compound (Fig. 1) was obtained in a few step synthesis starting from the reaction of diquinodithiin or 2,2'-dichloro-3,3'-diquinolinyl disulfide with p-chloroaniline. The obtained 6H-9-chloroquinobenzothiazine *via* thiazine ring formation (Pluta *et al.*, 2009) was next alkylated with phthalimidobutyl bromide, hydrolyzed with hydrazine and acetylated with acetic anhydride (Pluta *et al.*, 2012). The structure elucidation was based on the ^1^H NMR spectrum which did not take all the doubts away. These reactions may lead to alternative products (II-V) (Fig. 1) as the results of other ring closure reaction, the Smiles rearrangement, tautomeric forms and competitive N-alkylation. The X-ray analysis fully confirmed the proposed structure as [3,2-*b*], the chlorine atom in position 9 and the acetylaminobutyl substituent at the thiazine nitrogen atom N6. The tetracyclic ring system (the plane from C1 atom up to C12A atom) in title molecule is unexpectedly almost planar [r.m.s. deviation 0.110 (4) Å]) with the dihedral angle between the quinoline and the benzene ring of 178.3 (1)° and the angle between two halves of the thiazine ring of 173.4 (1)°. All the classical neuroleptic phenothiazines are folded along theN–S axis with the angle of 134.0–153.6 ° (Chu, 1988) and the title molecule is the first example of planar azaphenothiazine with the aminoalkyl group at the thiazine nitrogen atom. Other similar tetracyclic compounds with the thiazine ring, 6-benzyl-10-trifluoromethylquinobenzothiazine (Pluta *et al.*, 2012), 6-methyldihydroquinobenzothiazine (Luck *et al.*, 2003) and 5H-naphthobenzothiazine (Yoshida *et al.*, 1994) were also folded. Close to planar structure was 6H-8-trifluoromethylquinobenzothiazine (Pluta *et al.*, 2012). The C10A–S11–C11A and C5A–N6–C6A bond angles are quite large, 102.2 (2)° and 123.8 (3)° and enable the thiazine ring to adopt the flat conformation. Both the thiazine N6 and the amide N18 nitrogen atoms do not show pyramidality as the sum of C–N–X bond angles (X = C or H) is 360.1 (5)° and 360°, repectively. The side chain is not coplanar with the tetracyclic system. The torsion angles involving the butyl group (C14–C17) show the antiperiplanar arrangement of the carbon chain. The torsion angle C16–C17–N18–C19 [135.2 (4)°] describes the anticlinal arrangement of these atoms. In the crystal, molecules are arranged into stacks *via*π–π interactions with centroid-to-centroid distances in the range of 3.603 (2)–3.739 (2) Å and extending along the *b* crystallographic axis ( Fig. 3). N–H···O hydrogen bond (Table 1) connects adjacent molecules along the stacks via catemeric C(4) motif (Fig. 4). The significant antiproliferative and anticancer activities of the title molecule most probabably result from intercalation of specific DNA by the planar azaphenothiazine system.

### Experimental

The title compound was obtained in a few step synthesis starting from the reaction of diquinodithiin or 2,2'-dichloro-3,3'-diquinolinyl disulfide with p-chloroaniline (Jeleń *et al.*, 2009). The obtained 6H-9-chloroquinobenzothiazine was alkylated with phthalimidobutyl bromide in dry toluene in the presence of sodium hydride, hydrolyzed with hydrazine in ethanol and acetylated with acetican hydride in pyridine. The title compound has melting point 417-418 K (Pluta *et al.*, 2012). X-ray quality crystals were grown from chloroform-ethanol mixture by slow evaporation.

### Refinement

All H atoms were treated as riding atoms in geometrically calculated positions, with d(C–H) = 0.95, 0.99 and 0.98 Å for aromatic, methylene and methyl hydrogens, respectively, d(N–H) = 0.88 Å , and *U*_iso_(H) = *kU*_eq_(C,N), where *k* = 1.5 for the methyl group and *k* = 1.2 otherwise.

### Crystal data

**Table:**

C_21_H_20_ClN_3_OS	*F*(000) = 832
*M**_r_* = 397.92	*D*_x_ = 1.464 Mg m^−^^3^
Monoclinic, *P*2_1_/*c*	Mo *K*α radiation, λ = 0.71073 Å
Hall symbol: -P 2ybc	Cell parameters from 5229 reflections
*a* = 12.7800 (4) Å	θ = 2.9–24.7°
*b* = 4.9530 (11) Å	µ = 0.35 mm^−^^1^
*c* = 28.781 (2) Å	*T* = 100 K
β = 97.726 (5)°	Needle, yellow
*V* = 1805.3 (4) Å^3^	0.60 × 0.10 × 0.05 mm
*Z* = 4	

### Data collection

**Table:**

Nonius KappaCCD diffractometer upgraded with an APEXII detector	1987 reflections with *I* > 2σ(*I*)
Radiation source: fine-focus sealed tube	*R*_int_ = 0.121
Graphite monochromator	θ_max_ = 24.7°, θ_min_ = 3.1°
Detector resolution: 8.3 pixels mm^-1^	*h* = −15→15
ω scan	*k* = −5→5
17434 measured reflections	*l* = −32→33
3032 independent reflections	

### Refinement

**Table:**

Refinement on *F*^2^	Primary atom site location: structure-invariant direct methods
Least-squares matrix: full	Secondary atom site location: difference Fourier map
*R*[*F*^2^ > 2σ(*F*^2^)] = 0.067	Hydrogen site location: inferred from neighbouring sites
*wR*(*F*^2^) = 0.127	H-atom parameters constrained
*S* = 1.10	*w* = 1/[σ^2^(*F*_o_^2^) + (0.016*P*)^2^ + 1.5516*P*] where *P* = (*F*_o_^2^ + 2*F*_c_^2^)/3
3032 reflections	(Δ/σ)_max_ < 0.001
245 parameters	Δρ_max_ = 0.32 e Å^−^^3^
0 restraints	Δρ_min_ = −0.29 e Å^−^^3^

### Special details

**Table:**

Geometry. All e.s.d.'s (except the e.s.d. in the dihedral angle between two l.s. planes) are estimated using the full covariance matrix. The cell e.s.d.'s are taken into account individually in the estimation of e.s.d.'s in distances, angles and torsion angles; correlations between e.s.d.'s in cell parameters are only used when they are defined by crystal symmetry. An approximate (isotropic) treatment of cell e.s.d.'s is used for estimating e.s.d.'s involving l.s. planes.
Refinement. Refinement of *F*^2^ against ALL reflections. The weighted *R*-factor *wR* and goodness of fit *S* are based on *F*^2^, conventional *R*-factors *R* are based on *F*, with *F* set to zero for negative *F*^2^. The threshold expression of *F*^2^ > 2σ(*F*^2^) is used only for calculating *R*-factors(gt) *etc*. and is not relevant to the choice of reflections for refinement. *R*-factors based on *F*^2^ are statistically about twice as large as those based on *F*, and *R*- factors based on ALL data will be even larger.

### Fractional atomic coordinates and isotropic or equivalent isotropic displacement parameters (Å^2^)

**Table:**

	*x*	*y*	*z*	*U*_iso_*/*U*_eq_	
C1	1.1591 (3)	0.0335 (8)	0.38503 (15)	0.0286 (11)	
H1	1.1969	0.0022	0.4153	0.034*	
C2	1.1827 (3)	−0.1165 (8)	0.34779 (14)	0.0298 (11)	
H2	1.2360	−0.2513	0.3520	0.036*	
C3	1.1264 (3)	−0.0669 (8)	0.30318 (15)	0.0285 (11)	
H3	1.1426	−0.1689	0.2771	0.034*	
C4	1.0484 (3)	0.1260 (8)	0.29646 (14)	0.0252 (10)	
H4	1.0116	0.1557	0.2660	0.030*	
C4A	1.0226 (3)	0.2802 (8)	0.33455 (14)	0.0240 (10)	
C5A	0.9204 (3)	0.6158 (8)	0.36259 (14)	0.0249 (10)	
C6A	0.8031 (3)	0.9780 (8)	0.38605 (14)	0.0249 (10)	
C7	0.7205 (3)	1.1593 (8)	0.37258 (14)	0.0251 (10)	
H7	0.6895	1.1630	0.3407	0.030*	
C8	0.6820 (3)	1.3348 (8)	0.40429 (14)	0.0281 (11)	
H8	0.6289	1.4636	0.3939	0.034*	
C9	0.7229 (3)	1.3170 (8)	0.45101 (14)	0.0278 (11)	
C10	0.8017 (3)	1.1362 (8)	0.46564 (14)	0.0283 (11)	
H10	0.8280	1.1239	0.4980	0.034*	
C10A	0.8439 (3)	0.9705 (8)	0.43379 (14)	0.0258 (10)	
C11A	0.9741 (3)	0.5778 (8)	0.40921 (14)	0.0244 (10)	
C12	1.0516 (3)	0.3889 (8)	0.41680 (14)	0.0246 (10)	
H12	1.0871	0.3616	0.4476	0.030*	
C12A	1.0802 (3)	0.2326 (8)	0.37939 (14)	0.0243 (10)	
C14	0.8053 (3)	0.8667 (8)	0.30219 (13)	0.0257 (10)	
H14A	0.8611	0.8131	0.2833	0.031*	
H14B	0.7936	1.0632	0.2978	0.031*	
C15	0.7036 (3)	0.7195 (8)	0.28324 (13)	0.0252 (10)	
H15A	0.6482	0.7613	0.3031	0.030*	
H15B	0.7160	0.5222	0.2843	0.030*	
C16	0.6666 (3)	0.8062 (8)	0.23276 (13)	0.0272 (11)	
H16A	0.6422	0.9960	0.2327	0.033*	
H16B	0.7271	0.7982	0.2147	0.033*	
C17	0.5779 (3)	0.6321 (9)	0.20864 (14)	0.0316 (11)	
H17A	0.5948	0.4398	0.2154	0.038*	
H17B	0.5119	0.6750	0.2215	0.038*	
C19	0.5476 (3)	0.4691 (9)	0.12731 (15)	0.0290 (11)	
C20	0.5415 (3)	0.5422 (9)	0.07638 (14)	0.0362 (12)	
H20A	0.5999	0.4565	0.0631	0.054*	
H20B	0.5463	0.7387	0.0733	0.054*	
H20C	0.4742	0.4791	0.0595	0.054*	
N5	0.9431 (2)	0.4684 (6)	0.32683 (11)	0.0227 (8)	
N6	0.8438 (3)	0.8161 (6)	0.35178 (11)	0.0237 (8)	
N18	0.5615 (3)	0.6734 (7)	0.15804 (11)	0.0279 (9)	
H18	0.5607	0.8396	0.1472	0.033*	
O21	0.5405 (2)	0.2316 (6)	0.14005 (10)	0.0400 (8)	
S11	0.94961 (9)	0.7684 (2)	0.45749 (4)	0.0304 (3)	
Cl13	0.67276 (9)	1.5212 (2)	0.49241 (4)	0.0364 (3)	

### Atomic displacement parameters (Å^2^)

**Table:**

	*U*^11^	*U*^22^	*U*^33^	*U*^12^	*U*^13^	*U*^23^
C1	0.025 (3)	0.028 (3)	0.032 (3)	−0.002 (2)	0.003 (2)	0.005 (2)
C2	0.025 (3)	0.026 (3)	0.040 (3)	0.000 (2)	0.008 (2)	0.002 (2)
C3	0.030 (3)	0.022 (3)	0.035 (3)	−0.006 (2)	0.007 (2)	0.000 (2)
C4	0.027 (3)	0.023 (2)	0.026 (2)	−0.009 (2)	0.002 (2)	−0.001 (2)
C4A	0.025 (2)	0.016 (2)	0.032 (2)	−0.004 (2)	0.007 (2)	0.003 (2)
C5A	0.026 (3)	0.019 (2)	0.030 (2)	−0.002 (2)	0.005 (2)	0.002 (2)
C6A	0.023 (2)	0.023 (2)	0.029 (2)	−0.004 (2)	0.003 (2)	0.003 (2)
C7	0.027 (3)	0.024 (3)	0.025 (2)	−0.009 (2)	0.005 (2)	0.001 (2)
C8	0.026 (3)	0.022 (3)	0.037 (3)	−0.003 (2)	0.006 (2)	0.005 (2)
C9	0.030 (3)	0.021 (3)	0.034 (3)	−0.002 (2)	0.008 (2)	−0.003 (2)
C10	0.031 (3)	0.027 (3)	0.027 (2)	−0.005 (2)	0.001 (2)	0.001 (2)
C10A	0.024 (2)	0.024 (2)	0.029 (2)	−0.002 (2)	0.004 (2)	0.002 (2)
C11A	0.027 (3)	0.019 (2)	0.028 (2)	−0.004 (2)	0.007 (2)	0.0015 (19)
C12	0.024 (2)	0.021 (2)	0.029 (2)	−0.006 (2)	0.004 (2)	0.005 (2)
C12A	0.021 (2)	0.021 (2)	0.031 (2)	−0.003 (2)	0.005 (2)	0.003 (2)
C14	0.033 (3)	0.021 (2)	0.023 (2)	0.002 (2)	0.003 (2)	0.0061 (19)
C15	0.028 (3)	0.021 (2)	0.026 (2)	−0.003 (2)	0.001 (2)	0.0018 (19)
C16	0.032 (3)	0.020 (2)	0.032 (2)	0.000 (2)	0.009 (2)	−0.0005 (19)
C17	0.037 (3)	0.028 (3)	0.029 (2)	−0.002 (2)	0.001 (2)	0.008 (2)
C19	0.022 (2)	0.029 (3)	0.034 (3)	0.002 (2)	−0.003 (2)	0.000 (2)
C20	0.037 (3)	0.033 (3)	0.040 (3)	0.002 (2)	0.009 (2)	−0.011 (2)
N5	0.0211 (19)	0.018 (2)	0.029 (2)	0.0004 (17)	0.0033 (16)	0.0048 (16)
N6	0.027 (2)	0.021 (2)	0.0228 (19)	−0.0017 (17)	0.0043 (16)	0.0008 (16)
N18	0.035 (2)	0.018 (2)	0.029 (2)	−0.0034 (17)	−0.0004 (17)	0.0051 (16)
O21	0.050 (2)	0.0159 (18)	0.050 (2)	−0.0015 (16)	−0.0054 (16)	−0.0007 (15)
S11	0.0343 (7)	0.0290 (7)	0.0271 (6)	0.0036 (6)	0.0006 (5)	−0.0018 (5)
Cl13	0.0404 (7)	0.0309 (7)	0.0391 (7)	0.0040 (6)	0.0105 (6)	−0.0054 (5)

### Geometric parameters (Å, º)

**Table:**

C1—C2	1.371 (5)	C10A—S11	1.745 (4)
C1—C12A	1.405 (5)	C11A—C12	1.359 (5)
C1—H1	0.9500	C11A—S11	1.743 (4)
C2—C3	1.406 (5)	C12—C12A	1.413 (5)
C2—H2	0.9500	C12—H12	0.9500
C3—C4	1.376 (5)	C14—N6	1.468 (4)
C3—H3	0.9500	C14—C15	1.526 (5)
C4—C4A	1.411 (5)	C14—H14A	0.9900
C4—H4	0.9500	C14—H14B	0.9900
C4A—N5	1.374 (5)	C15—C16	1.528 (5)
C4A—C12A	1.417 (5)	C15—H15A	0.9900
C5A—N5	1.325 (5)	C15—H15B	0.9900
C5A—N6	1.399 (5)	C16—C17	1.515 (5)
C5A—C11A	1.435 (5)	C16—H16A	0.9900
C6A—C7	1.400 (5)	C16—H16B	0.9900
C6A—C10A	1.403 (5)	C17—N18	1.458 (5)
C6A—N6	1.423 (5)	C17—H17A	0.9900
C7—C8	1.397 (5)	C17—H17B	0.9900
C7—H7	0.9500	C19—O21	1.239 (5)
C8—C9	1.378 (5)	C19—N18	1.340 (5)
C8—H8	0.9500	C19—C20	1.502 (5)
C9—C10	1.371 (5)	C20—H20A	0.9800
C9—Cl13	1.749 (4)	C20—H20B	0.9800
C10—C10A	1.392 (5)	C20—H20C	0.9800
C10—H10	0.9500	N18—H18	0.8800
			
C2—C1—C12A	121.4 (4)	C1—C12A—C4A	119.9 (4)
C2—C1—H1	119.3	C12—C12A—C4A	116.6 (4)
C12A—C1—H1	119.3	N6—C14—C15	115.1 (3)
C1—C2—C3	118.7 (4)	N6—C14—H14A	108.5
C1—C2—H2	120.7	C15—C14—H14A	108.5
C3—C2—H2	120.7	N6—C14—H14B	108.5
C4—C3—C2	121.4 (4)	C15—C14—H14B	108.5
C4—C3—H3	119.3	H14A—C14—H14B	107.5
C2—C3—H3	119.3	C14—C15—C16	110.2 (3)
C3—C4—C4A	120.5 (4)	C14—C15—H15A	109.6
C3—C4—H4	119.8	C16—C15—H15A	109.6
C4A—C4—H4	119.8	C14—C15—H15B	109.6
N5—C4A—C4	119.1 (4)	C16—C15—H15B	109.6
N5—C4A—C12A	122.8 (4)	H15A—C15—H15B	108.1
C4—C4A—C12A	118.1 (4)	C17—C16—C15	113.1 (3)
N5—C5A—N6	115.9 (4)	C17—C16—H16A	109.0
N5—C5A—C11A	121.8 (4)	C15—C16—H16A	109.0
N6—C5A—C11A	122.2 (4)	C17—C16—H16B	109.0
C7—C6A—C10A	117.1 (4)	C15—C16—H16B	109.0
C7—C6A—N6	120.1 (4)	H16A—C16—H16B	107.8
C10A—C6A—N6	122.8 (4)	N18—C17—C16	112.1 (3)
C8—C7—C6A	122.5 (4)	N18—C17—H17A	109.2
C8—C7—H7	118.8	C16—C17—H17A	109.2
C6A—C7—H7	118.8	N18—C17—H17B	109.2
C9—C8—C7	118.5 (4)	C16—C17—H17B	109.2
C9—C8—H8	120.8	H17A—C17—H17B	107.9
C7—C8—H8	120.8	O21—C19—N18	122.0 (4)
C10—C9—C8	120.5 (4)	O21—C19—C20	121.4 (4)
C10—C9—Cl13	119.3 (3)	N18—C19—C20	116.5 (4)
C8—C9—Cl13	120.1 (3)	C19—C20—H20A	109.5
C9—C10—C10A	121.1 (4)	C19—C20—H20B	109.5
C9—C10—H10	119.5	H20A—C20—H20B	109.5
C10A—C10—H10	119.5	C19—C20—H20C	109.5
C10—C10A—C6A	120.3 (4)	H20A—C20—H20C	109.5
C10—C10A—S11	115.4 (3)	H20B—C20—H20C	109.5
C6A—C10A—S11	124.3 (3)	C5A—N5—C4A	118.8 (3)
C12—C11A—C5A	119.2 (4)	C5A—N6—C6A	123.8 (3)
C12—C11A—S11	116.7 (3)	C5A—N6—C14	118.1 (3)
C5A—C11A—S11	124.1 (3)	C6A—N6—C14	118.2 (3)
C11A—C12—C12A	120.8 (4)	C19—N18—C17	122.8 (3)
C11A—C12—H12	119.6	C19—N18—H18	118.6
C12A—C12—H12	119.6	C17—N18—H18	118.6
C1—C12A—C12	123.5 (4)	C11A—S11—C10A	102.2 (2)

### Hydrogen-bond geometry (Å, º)

**Table:**

*D*—H···*A*	*D*—H	H···*A*	*D*···*A*	*D*—H···*A*
N18—H18···O21^i^	0.88	1.97	2.819 (4)	163

Symmetry code: (i) *x*, *y*+1, *z*.
